# (*E*)-3-[(2-Methyl-4-nitro­phen­yl)imino­meth­yl]-1-benzothio­phene

**DOI:** 10.1107/S1600536812000190

**Published:** 2012-01-11

**Authors:** Hasan Inaç, Necmi Dege, Sümeyye Gümüş, Erbil Ağar, Mustafa Serkan Soylu

**Affiliations:** a Kırıkkale University, Faculty of Education, Department of Elementary Education, Science Teacher Training Programme, 71451 Kırıkkale, Turkey; b Ondokuz Mayıs University, Arts and Sciences Faculty, Department of Physics, 55139 Samsun, Turkey; c Ondokuz Mayıs University, Arts and Sciences Faculty, Department of Chemistry, 55139 Samsun, Turkey; d Giresun University, Faculty of Arts and Sciences, Department of Physics, 28100 Giresun, Turkey

## Abstract

In the title conpound, C_16_H_12_N_2_O_2_S, the 1-benzothio­phene residue and the substituted benzene ring are oriented at a dihedral angle of 53.36 (6)°. The mol­ecular conformation features a short C—H⋯N contact. There are no significant inter­molecular contacts.

## Related literature

For the biological activity of Schiff bases, see: Barton *et al.* (1979 ▶); Ingold (1969 ▶); Layer (1963 ▶). For industrial applications of Schiff bases, see: Taggi *et al.* (2002 ▶). For chemical properties of Schiff bases, see: Aydoğan *et al.* (2001 ▶). For related structures, see: Ağar *et al.* (2010 ▶); Ceylan *et al.* (2011 ▶); Dege *et al.* (2006 ▶); Demirtaş *et al.* (2009 ▶); Tecer *et al.* (2010 ▶). 
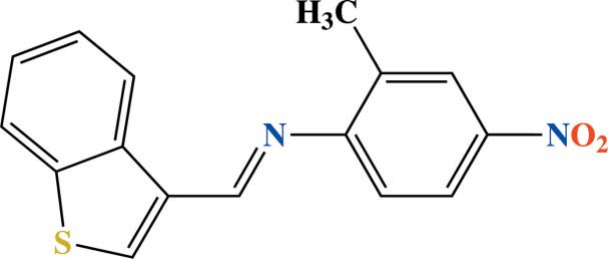


## Experimental

### 

#### Crystal data

C_16_H_12_N_2_O_2_S
*M* *_r_* = 296.34
Monoclinic, 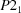

*a* = 7.6224 (4) Å
*b* = 7.9139 (4) Å
*c* = 11.7536 (5) Å
β = 91.341 (4)°
*V* = 708.82 (6) Å^3^
*Z* = 2
Mo *K*α radiation
μ = 0.23 mm^−1^
*T* = 296 K
0.17 × 0.15 × 0.12 mm

#### Data collection

Oxford Diffraction SuperNova Single source at offset diffractometer with an Eos detector
Absorption correction: multi-scan (*CrysAlis PRO*; Oxford Diffraction, 2009 ▶) *T*_min_ = 0.563, *T*_max_ = 1.000
2851 measured reflections
2108 independent reflections
1870 reflections with *I* > 2σ(*I*)
*R*_int_ = 0.013

#### Refinement

*R*[*F* ^2^ > 2σ(*F* ^2^)] = 0.037
*wR*(*F* ^2^) = 0.089
*S* = 1.05
2108 reflections
191 parameters
1 restraint
H-atom parameters constrained
Δρ_max_ = 0.15 e Å^−3^
Δρ_min_ = −0.14 e Å^−3^
Absolute structure: Flack (1983 ▶), 572 Friedel pairs
Flack parameter: −0.07 (11)

### 

Data collection: *CrysAlis PRO* (Oxford Diffraction, 2009 ▶); cell refinement: *CrysAlis PRO*; data reduction: *CrysAlis PRO*; program(s) used to solve structure: *WinGX* (Farrugia, 1997 ▶) and *SHELXS97* (Sheldrick, 2008 ▶); program(s) used to refine structure: *SHELXL97* (Sheldrick, 2008 ▶); molecular graphics: *OLEX2* (Dolomanov *et al.*, 2009 ▶) and *ORTEP-3 for Windows* (Farrugia, 1997 ▶); software used to prepare material for publication: *OLEX2*, *WinGX* (Farrugia, 1999 ▶) and *PLATON* (Spek, 2009 ▶).

## Supplementary Material

Crystal structure: contains datablock(s) I, global. DOI: 10.1107/S1600536812000190/bt5774sup1.cif

Structure factors: contains datablock(s) I. DOI: 10.1107/S1600536812000190/bt5774Isup2.hkl

Supplementary material file. DOI: 10.1107/S1600536812000190/bt5774Isup3.cml

Additional supplementary materials:  crystallographic information; 3D view; checkCIF report

## Figures and Tables

**Table 1:** Hydrogen-bond geometry (Å, °)

*D*—H⋯*A*	*D*—H	H⋯*A*	*D*⋯*A*	*D*—H⋯*A*
C5—H5⋯N1	0.93	2.54	3.093 (4)	118

**Fig. 1.**
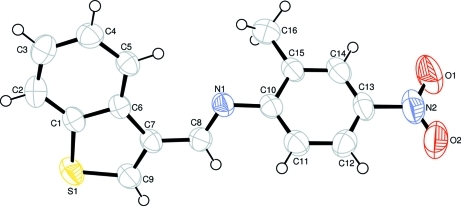
The asymmetric unit of the title compound, showing the atomic numbering scheme. Displacement ellipsoids are drawn at the 50% probability level.

## supplementary crystallographic information

### Comment

Schiff bases, *i.e.*, compounds having a double C=N bond, are used as starting materials in the synthesis of important drugs, such as antibiotics and antiallergic, antiphlogistic, and antitumor substances (Barton *et al.*, 1979; Layer, 1963; Ingold 1969). On the industrial scale, they have a wide range of applications, such as dyes and pigments (Taggi *et al.*, 2002). Schiff bases have also been employed as ligands for the complexation of metal ions (Aydoğan *et al.*, 2001).

The dihedral angle between the C10—C15 benzene and the C1—C9/S1 benzothiophene ring is 53.36 (6)°. The length of the C8=N1 double bond is 1.271 (3) Å, slightly shorter than standard 1.28 Å value of a C=N double bond and consistent with related structures (Ağar *et al.*, 2010; Tecer *et al.*, 2010; Ceylan *et al.* 2011; Demirtaş *et al.*, 2009).

In the compound, N—O bond distances are 1.218 (4) Å for N2—O1 and N2—O2. The O1—N2—O2 bond angle is 123.4 (3)°. The C1—S1 and C9—S1 bond distances are 1.738 (3) Å and 1.709 (3) Å, respectively. The C—S bond distances are compatible with the literature (Dege *et al.* 2006; Demirtaş *et al.*, 2009).

### Experimental

The compound (*E*)-1-(1-benzothiophen-3-yl)-*N*-(2-methyl-4-nitrophenyl)methanimine was prepared by refluxing a mixture of a solution containing 1-benzothiophene-3-carbaldehyde (0.0102 g 0.063 mmol) in 20 ml ethanol and a solution containing 2-Methyl-4-nitroaniline (0.0095 g 0.063 mmol) in 20 ml ethanol. The reaction mixture was stirred for 1 h under reflux. The crystals of(*E*)-4-((benzo[*b*]thiophen-3-ylmethylene)amino)-3-methylbenzoic acid suitable for X-ray analysis were obtained from ethylalcohol by slow evaporation (yield = 63%; m.p: 153–155°C).

### Refinement

All hydrogen atoms were positioned geometrically with C—H = 0.93 Å and *U*_iso_(H)=1.2*U*_eq_(C) or with C—H = 0.960 Å and 1.5*U*_eq_(C_methyl_).

### Crystal data

**Table:**

C_16_H_12_N_2_O_2_S	*F*(000) = 308
*M**_r_* = 296.34	*D*_x_ = 1.388 Mg m^−^^3^
Monoclinic, *P*2_1_	Mo *K*α radiation, λ = 0.7107 Å
Hall symbol: P 2yb	Cell parameters from 1356 reflections
*a* = 7.6224 (4) Å	θ = 3.2–27.7°
*b* = 7.9139 (4) Å	µ = 0.23 mm^−^^1^
*c* = 11.7536 (5) Å	*T* = 296 K
β = 91.341 (4)°	Prism, brown
*V* = 708.82 (6) Å^3^	0.17 × 0.15 × 0.12 mm
*Z* = 2	

### Data collection

**Table:**

Oxford Diffraction SuperNova Single source at offset diffractometer with an Eos detector	2108 independent reflections
Radiation source: SuperNova (Mo) X-ray Source	1870 reflections with *I* > 2σ(*I*)
mirror	*R*_int_ = 0.013
Detector resolution: 16.0454 pixels mm^-1^	θ_max_ = 27.8°, θ_min_ = 3.2°
ω scans	*h* = −4→9
Absorption correction: multi-scan (*CrysAlis PRO*; Oxford Diffraction, 2009)	*k* = −10→5
*T*_min_ = 0.563, *T*_max_ = 1.000	*l* = −15→14
2851 measured reflections	

### Refinement

**Table:**

Refinement on *F*^2^	Secondary atom site location: difference Fourier map
Least-squares matrix: full	Hydrogen site location: inferred from neighbouring sites
*R*[*F*^2^ > 2σ(*F*^2^)] = 0.037	H-atom parameters constrained
*wR*(*F*^2^) = 0.089	*w* = 1/[σ^2^(*F*_o_^2^) + (0.0369*P*)^2^ + 0.1426*P*] where *P* = (*F*_o_^2^ + 2*F*_c_^2^)/3
*S* = 1.05	(Δ/σ)_max_ < 0.001
2108 reflections	Δρ_max_ = 0.15 e Å^−^^3^
191 parameters	Δρ_min_ = −0.14 e Å^−^^3^
1 restraint	Absolute structure: Flack (1983), 572 Friedel pairs
Primary atom site location: structure-invariant direct methods	Flack parameter: −0.07 (11)

### Special details

**Table:**

Experimental. *CrysAlis PRO* (Oxford Diffraction, 2009)
Geometry. All e.s.d.'s (except the e.s.d. in the dihedral angle between two l.s. planes) are estimated using the full covariance matrix. The cell e.s.d.'s are taken into account individually in the estimation of e.s.d.'s in distances, angles and torsion angles; correlations between e.s.d.'s in cell parameters are only used when they are defined by crystal symmetry. An approximate (isotropic) treatment of cell e.s.d.'s is used for estimating e.s.d.'s involving l.s. planes.
Refinement. Refinement of *F*^2^ against ALL reflections. The weighted *R*-factor *wR* and goodness of fit *S* are based on *F*^2^, conventional *R*-factors *R* are based on *F*, with *F* set to zero for negative *F*^2^. The threshold expression of *F*^2^ > σ(*F*^2^) is used only for calculating *R*-factors(gt) *etc*. and is not relevant to the choice of reflections for refinement. *R*-factors based on *F*^2^ are statistically about twice as large as those based on *F*, and *R*- factors based on ALL data will be even larger.

### Fractional atomic coordinates and isotropic or equivalent isotropic displacement parameters (Å^2^)

**Table:**

	*x*	*y*	*z*	*U*_iso_*/*U*_eq_	
C1	0.7745 (4)	0.7800 (4)	0.3597 (2)	0.0487 (7)	
C2	0.9349 (4)	0.8478 (4)	0.3262 (3)	0.0621 (8)	
H2	0.9650	0.8497	0.2500	0.075*	
C3	1.0455 (4)	0.9109 (5)	0.4081 (3)	0.0662 (9)	
H3	1.1529	0.9561	0.3876	0.079*	
C4	0.9999 (4)	0.9087 (5)	0.5225 (3)	0.0591 (8)	
H4	1.0768	0.9545	0.5768	0.071*	
C5	0.8438 (4)	0.8401 (4)	0.5565 (2)	0.0491 (7)	
H5	0.8165	0.8377	0.6332	0.059*	
C6	0.7263 (4)	0.7740 (3)	0.4744 (2)	0.0444 (6)	
C7	0.5540 (3)	0.6999 (5)	0.4864 (2)	0.0488 (6)	
C8	0.4620 (3)	0.6719 (4)	0.5914 (2)	0.0512 (7)	
H8	0.3469	0.6330	0.5868	0.061*	
C9	0.4821 (4)	0.6556 (4)	0.3838 (2)	0.0585 (8)	
H9	0.3708	0.6083	0.3759	0.070*	
C10	0.4313 (3)	0.6683 (4)	0.7870 (2)	0.0497 (7)	
C11	0.2601 (4)	0.7284 (4)	0.7959 (2)	0.0597 (8)	
H11	0.2086	0.7888	0.7360	0.072*	
C12	0.1660 (4)	0.6983 (5)	0.8939 (2)	0.0639 (8)	
H12	0.0516	0.7373	0.9004	0.077*	
C13	0.2467 (4)	0.6093 (4)	0.9806 (2)	0.0603 (9)	
C14	0.4166 (4)	0.5529 (5)	0.9747 (2)	0.0614 (8)	
H14	0.4677	0.4944	1.0355	0.074*	
C15	0.5110 (4)	0.5831 (4)	0.8783 (2)	0.0539 (8)	
C16	0.6974 (4)	0.5212 (6)	0.8702 (3)	0.0789 (11)	
H16A	0.7464	0.5045	0.9453	0.118*	
H16B	0.7657	0.6034	0.8307	0.118*	
H16C	0.6986	0.4162	0.8293	0.118*	
N1	0.5308 (3)	0.6980 (4)	0.68930 (16)	0.0527 (6)	
N2	0.1479 (5)	0.5769 (5)	1.0843 (3)	0.0851 (10)	
O1	0.2209 (5)	0.5011 (5)	1.1621 (2)	0.1246 (13)	
O2	−0.0032 (4)	0.6264 (5)	1.0876 (2)	0.1153 (14)	
S1	0.61283 (11)	0.69395 (13)	0.27057 (6)	0.0641 (2)	

### Atomic displacement parameters (Å^2^)

**Table:**

	*U*^11^	*U*^22^	*U*^33^	*U*^12^	*U*^13^	*U*^23^
C1	0.0627 (17)	0.0399 (16)	0.0435 (14)	0.0049 (13)	0.0028 (12)	0.0020 (13)
C2	0.077 (2)	0.055 (2)	0.0559 (18)	−0.0021 (18)	0.0215 (15)	−0.0005 (16)
C3	0.0635 (19)	0.060 (2)	0.075 (2)	−0.0075 (17)	0.0136 (17)	0.0057 (18)
C4	0.0594 (17)	0.057 (2)	0.061 (2)	−0.0096 (16)	−0.0056 (15)	0.0006 (16)
C5	0.0591 (17)	0.0436 (16)	0.0445 (15)	−0.0019 (14)	−0.0013 (12)	0.0024 (14)
C6	0.0537 (15)	0.0366 (15)	0.0431 (14)	0.0046 (12)	0.0027 (11)	0.0028 (12)
C7	0.0583 (15)	0.0451 (16)	0.0430 (12)	−0.0049 (15)	−0.0015 (10)	0.0052 (16)
C8	0.0521 (14)	0.0483 (18)	0.0533 (15)	−0.0074 (15)	0.0006 (11)	0.0051 (16)
C9	0.0659 (18)	0.059 (2)	0.0505 (15)	−0.0105 (16)	−0.0039 (13)	0.0039 (15)
C10	0.0571 (15)	0.0471 (18)	0.0447 (13)	−0.0116 (15)	−0.0014 (11)	−0.0015 (14)
C11	0.0644 (18)	0.062 (2)	0.0524 (15)	−0.0003 (16)	−0.0018 (13)	0.0056 (16)
C12	0.0562 (16)	0.071 (2)	0.0654 (17)	−0.002 (2)	0.0102 (13)	−0.013 (2)
C13	0.074 (2)	0.067 (2)	0.0397 (15)	−0.0203 (16)	0.0091 (14)	−0.0096 (15)
C14	0.077 (2)	0.063 (2)	0.0440 (16)	−0.0119 (17)	−0.0038 (15)	−0.0017 (15)
C15	0.0631 (18)	0.058 (2)	0.0409 (15)	−0.0080 (14)	−0.0032 (13)	0.0020 (14)
C16	0.069 (2)	0.101 (3)	0.067 (2)	0.011 (2)	−0.0075 (17)	0.014 (2)
N1	0.0554 (12)	0.0593 (15)	0.0433 (11)	−0.0099 (15)	0.0006 (9)	0.0055 (15)
N2	0.100 (2)	0.103 (3)	0.0538 (18)	−0.034 (2)	0.0222 (17)	−0.0200 (18)
O1	0.149 (3)	0.169 (4)	0.0565 (16)	−0.017 (3)	0.0217 (17)	0.029 (2)
O2	0.096 (2)	0.175 (4)	0.0766 (17)	−0.027 (2)	0.0362 (15)	−0.0328 (19)
S1	0.0847 (5)	0.0675 (5)	0.0399 (3)	−0.0104 (5)	−0.0024 (3)	−0.0013 (4)

### Geometric parameters (Å, °)

**Table:**

C1—C2	1.401 (4)	C10—C11	1.395 (4)
C1—C6	1.406 (3)	C10—C15	1.395 (4)
C1—S1	1.738 (3)	C10—N1	1.411 (3)
C2—C3	1.359 (5)	C11—C12	1.391 (4)
C2—H2	0.9300	C11—H11	0.9300
C3—C4	1.397 (5)	C12—C13	1.373 (5)
C3—H3	0.9300	C12—H12	0.9300
C4—C5	1.376 (4)	C13—C14	1.373 (5)
C4—H4	0.9300	C13—N2	1.470 (4)
C5—C6	1.403 (4)	C14—C15	1.377 (4)
C5—H5	0.9300	C14—H14	0.9300
C6—C7	1.448 (4)	C15—C16	1.508 (4)
C7—C9	1.359 (3)	C16—H16A	0.9600
C7—C8	1.450 (3)	C16—H16B	0.9600
C8—N1	1.271 (3)	C16—H16C	0.9600
C8—H8	0.9300	N2—O1	1.218 (4)
C9—S1	1.709 (3)	N2—O2	1.218 (4)
C9—H9	0.9300		
			
C2—C1—C6	122.1 (3)	C11—C10—N1	121.7 (3)
C2—C1—S1	126.3 (2)	C15—C10—N1	118.3 (3)
C6—C1—S1	111.6 (2)	C12—C11—C10	120.3 (3)
C3—C2—C1	118.3 (3)	C12—C11—H11	119.8
C3—C2—H2	120.9	C10—C11—H11	119.8
C1—C2—H2	120.9	C13—C12—C11	118.2 (3)
C2—C3—C4	120.8 (3)	C13—C12—H12	120.9
C2—C3—H3	119.6	C11—C12—H12	120.9
C4—C3—H3	119.6	C12—C13—C14	122.4 (3)
C5—C4—C3	121.4 (3)	C12—C13—N2	118.4 (3)
C5—C4—H4	119.3	C14—C13—N2	119.2 (3)
C3—C4—H4	119.3	C13—C14—C15	119.8 (3)
C4—C5—C6	119.3 (3)	C13—C14—H14	120.1
C4—C5—H5	120.3	C15—C14—H14	120.1
C6—C5—H5	120.3	C14—C15—C10	119.3 (3)
C5—C6—C1	118.1 (3)	C14—C15—C16	120.5 (3)
C5—C6—C7	130.5 (2)	C10—C15—C16	120.1 (3)
C1—C6—C7	111.4 (2)	C15—C16—H16A	109.5
C9—C7—C6	111.4 (2)	C15—C16—H16B	109.5
C9—C7—C8	121.5 (3)	H16A—C16—H16B	109.5
C6—C7—C8	127.0 (2)	C15—C16—H16C	109.5
N1—C8—C7	123.2 (2)	H16A—C16—H16C	109.5
N1—C8—H8	118.4	H16B—C16—H16C	109.5
C7—C8—H8	118.4	C8—N1—C10	119.4 (2)
C7—C9—S1	114.5 (2)	O1—N2—O2	123.4 (3)
C7—C9—H9	122.7	O1—N2—C13	118.3 (4)
S1—C9—H9	122.7	O2—N2—C13	118.3 (4)
C11—C10—C15	119.9 (2)	C9—S1—C1	91.03 (13)
			
C6—C1—C2—C3	−0.6 (5)	C10—C11—C12—C13	0.3 (5)
S1—C1—C2—C3	179.6 (3)	C11—C12—C13—C14	1.4 (5)
C1—C2—C3—C4	−0.3 (5)	C11—C12—C13—N2	−179.9 (3)
C2—C3—C4—C5	1.2 (5)	C12—C13—C14—C15	−1.0 (5)
C3—C4—C5—C6	−1.3 (5)	N2—C13—C14—C15	−179.7 (3)
C4—C5—C6—C1	0.3 (4)	C13—C14—C15—C10	−1.2 (5)
C4—C5—C6—C7	−178.2 (3)	C13—C14—C15—C16	−179.7 (3)
C2—C1—C6—C5	0.6 (4)	C11—C10—C15—C14	2.8 (5)
S1—C1—C6—C5	−179.6 (2)	N1—C10—C15—C14	179.9 (3)
C2—C1—C6—C7	179.4 (3)	C11—C10—C15—C16	−178.7 (3)
S1—C1—C6—C7	−0.8 (3)	N1—C10—C15—C16	−1.6 (5)
C5—C6—C7—C9	178.3 (3)	C7—C8—N1—C10	179.7 (3)
C1—C6—C7—C9	−0.3 (4)	C11—C10—N1—C8	−47.3 (5)
C5—C6—C7—C8	−1.9 (5)	C15—C10—N1—C8	135.7 (3)
C1—C6—C7—C8	179.5 (3)	C12—C13—N2—O1	−178.5 (3)
C9—C7—C8—N1	173.7 (3)	C14—C13—N2—O1	0.3 (5)
C6—C7—C8—N1	−6.1 (6)	C12—C13—N2—O2	1.9 (5)
C6—C7—C9—S1	1.3 (4)	C14—C13—N2—O2	−179.3 (3)
C8—C7—C9—S1	−178.5 (3)	C7—C9—S1—C1	−1.5 (3)
C15—C10—C11—C12	−2.4 (5)	C2—C1—S1—C9	−178.9 (3)
N1—C10—C11—C12	−179.4 (3)	C6—C1—S1—C9	1.3 (2)

### Hydrogen-bond geometry (Å, °)

**Table:**

*D*—H···*A*	*D*—H	H···*A*	*D*···*A*	*D*—H···*A*
C5—H5···N1	0.93	2.54	3.093 (4)	118.
